# Incidence and treatment outcomes of secondary epiretinal membrane following intravitreal injection for diabetic macular edema

**DOI:** 10.1038/s41598-020-57509-6

**Published:** 2020-01-17

**Authors:** Yong Koo Kang, Han Sang Park, Dong Ho Park, Jae Pil Shin

**Affiliations:** Department of Ophthalmology, Kyungpook National University School of Medicine, Daegu, 41944 Korea

**Keywords:** Retinal diseases, Outcomes research

## Abstract

The purpose of this study was to investigate the incidence of secondary epiretinal membrane (ERM) after intravitreal injection and the effect of ERM on visual acuity and central macular thickness (CMT) in patients with diabetic macular edema (DME). We included 147 eyes of 95 patients over 18 years old who were diagnosed with DME from 2012 to 2016, treated with intravitreal injection, and followed-up more than 24 months. Mean CMT in the ERM group was significantly thicker than in the non-ERM group after 9, 12, 18, and 24 months. Secondary ERM developed in 9.5% of patients during follow-up. Compared to other agents, the incidence of secondary ERM was significantly higher after intravitreal injection of dexamethasone implant. Among patients in the ERM group, the mean decrease of CMT between pre-injection and 2 weeks post-injection was significantly less after secondary ERM formation than before ERM formation. Secondary ERM formation was significantly associated with the number of intravitreal injections and the use of dexamethasone implant. Therefore, secondary ERM develops more frequently as the number of intravitreal injections increases and after intravitreal dexamethasone implant injection. The therapeutic effects of intravitreal injections for DME patients decrease after secondary ERM formation.

## Introduction

Diabetic macular edema (DME) remains the most common cause of visual loss among diabetic retinopathy (DR) patients^1^. DME occurs following the breakdown of the blood–retinal barrier due to damage to vascular endothelial cell tight junctions and the loss of pericytes in retinal capillaries. DME is also caused by up regulation of increased inflammatory cytokines, such as chemokines, interleukin-6, interleukin-8, prostaglandins, and vascular endothelial growth factor (VEGF) induced by retinal tissue hypoxia^1–3^. Thus, intravitreal injections of anti-VEGF and steroids have been considered the standard management of DME and have been shown to improve clinical outcomes^4–6^.

Epiretinal membrane (ERM) is a pathology caused by a fibrocellular proliferation on the internal limiting membrane (ILM) followed by a cellular contraction and can occur secondary to DR^7^. Glial cells in ERM produce various cytokines and growth factors, which stimulate pathogenic neovascularization mediated by VEGF^8^. VEGF and its receptors are localized to both vascular and avascular ERM, and VEGF may contribute to the progression of DME^9^. Moreover, ERM may act as a physical barrier and decrease drug penetration after intravitreal injections of anti-VEGF or steroids in DME treatment^10^.

We investigated the incidence of secondary ERM and the effect of ERM on visual acuity and central macular thickness (CMT) among DME patients who received intravitreal injections. We also analyzed parameters contributing to secondary ERM formation following intravitreal injection.

## Results

### Demographics of patient

One hundred thirty-four patients were initially enrolled, but 39 patients were excluded. Thus, a total of 95 patients (147 eyes) were enrolled in this study. The demographic and clinical characteristics of all patients are summarized in Table 1.

**Table 1:** Clinical characteristics of patients who received intravitreal injections for diabetic macular edema.

Characteristics	Value	*P* value
Number of eyes, n (%)		
OD	75 (51.0%)	
OS	72 (49.0%)	
Sex, n (%)		
Male	46 (48.4%)	
Female	49 (51.6%)	
Age, years	59.7 ± 12.7	
Duration of diabetes mellitus, years	15.3 ± 8.6	
DM with HTN	35 (36.8%)	
DM with CKD	18 (18.9%)	
DM with Hyperlipidemia	12 (12.6%)	
LogMAR BCVA at baseline	0.540 ± 0.355	
Intraocular pressure, mmHg	15.6 ± 3.4	
Lens status, n (%)		
Phakia	113 (76.9%)	
Pseudophakia	34 (23.1%)	
Stage of diabetic retinopathy, n (%)		
Moderate NPDR	3 (0.02%)	
Severe NPDR	89 (60.5%)	
PDR	55 (37.4%)	
Type of diabetic macular edema in OCT, n (%)		
Diffuse macular edema	48 (32.7%)	
Cystoids macular edema	46 (31.3%)	
Serous retinal detachment	53 (36.0%)	
Previous panretinal photocoagulation, n (%)	80 (54.4%)	
Follow up periods, months	42.9 ± 18.7	
Mean number of intravitreal injection	5.0 ± 3.7	
ERM group (total)	7.1 ± 4.8	0.007*
Non-ERM group (total)	4.7 ± 3.1	
Incidence of ERM formation, n (%)	14 (9.5%)	
Mean time of ERM formation, months	19.3 ± 12.4	
Anti-VEGF treatment, %		
ERM group	53.8%	0.057*
Non-ERM group	75.4%	

Values are presented as the mean ± standard deviations.

DM: diabetes mellitus; HTN: hypertension; CKD: chronic kidney disease; BCVA: best-corrected visual acuity; ERM: epiretinal membrane; OCT: optical coherent tomography; NPDR: non-proliferative diabetic retinopathy; PDR: proliferative diabetic retinopathy; VEGF: vascular endothelial growth factor; ^*^Student t-test.

Among the 95 patients (147 eyes), 46 were male, and 49 were female. The mean age of all patients was 59.7 ± 12.7 years, and the duration of diabetes mellitus was 15.3 ± 8.6 years. Thirty-five patients had hypertension, 18 had chronic kidney disease, and 12 had hyperlipidemia. The mean logarithm of minimal angle of resolution (LogMAR) best corrected visual acuity (BCVA) was 0.540 ± 0.355, and the mean intraocular pressure was 15.6 ± 3.4 mmHg at the initial visit. Three eyes had moderate non-proliferative diabetic retinopathy (NPDR), 89 eyes had severe NPDR and 55 eyes had proliferative diabetic retinopathy (PDR). Moreover, 34 of 147 eyes were pseudophakic, and 80 eyes had received previous panretinal photocoagulation. The mean follow-up duration among all patients was 42.9 ± 18.7 months, and the mean number of intravitreal injections was 5.0 ± 3.7. The mean number of intravitreal injections among patients in the ERM group was 7.1 ± 4.8, which was significantly higher than 4.7 ± 3.1 for the non-ERM group (*P* = 0.007). The proportion of anti-VEGF intravitreal injections in the ERM group was 53.8%, which was lower than the 75.4% for the non-ERM group; however, this difference was not statistically significant (*P* = 0.057).

### Incidence of ERM and changes of BCVA and CMT

Fourteen eyes (9.5%) developed secondary ERM during follow-up, and the mean time to ERM formation was 19.3 ± 12.4 months (Table 1). According to the intravitreal injection agent, 7 of 108 eyes (6.5%) using anti-VEGF, 1 of 14 eyes (7.1%) using triamcinolone, and 6 of 25 eyes (24.0%) using dexamethasone implant developed secondary ERM. Compared to other agents, the incidence of ERM was significantly higher among patients who received dexamethasone implant injections (*P* = 0.025). Seven of 89 eyes (7.9%) with severe NPDR and 7 of 55 eyes (12.7%) with PDR developed secondary ERM and 8 of 80 eyes (10.0%) who previously received panretinal photocoagulation also developed secondary ERM during follow-up periods. According to the type of DME in optical coherent tomography (OCT), 2 of 48 eyes (4.2%) with diffuse macular edema, 8 of 46 eyes (17.4%) with cystoids macular edema, and 4 of 53 eyes (7.5%) of DME with serous retinal detachment developed secondary ERM. Compared to other types, the incidence of ERM was higher among patients with cystoids macular edema, however, this difference was not statistically significant (*P* = 0.077) (Table 2).

**Table 2:** The incidence of epiretinal membrane (ERM) formation according to clinical factors of diabetic retinopathy.

Incidence of ERM	Value	*P* value
According to drugs, n (%)		
Anti-VEGF	7 (6.5%)	0.025^†^
Dexamethasone implant	6 (24.0%)	
Triamcinolone	1 (7.1%)	
Stage of diabetic retinopathy, n (%)		
Moderate NPDR	0 (0.0%)	0.539^†^
Severe NPDR	7 (7.9%)	
PDR	7 (12.7%)	
History of panretinal photocoagulation, n (%)		
Yes	8 (10.0%)	0.790*
No	6 (9.0%)	
Type of diabetic macular edema, n (%)		
Diffuse macular edema	2 (4.2%)	0.077^†^
Cystoids macular edema	8 (17.4%)	
Serous retinal detachment	4 (7.5%)	

ERM: epiretinal membrane; NPDR: non-proliferative diabetic retinopathy; VEGF: vascular endothelial growth factor; *Student t-test, ^†^One-way analysis of variance.

**Figure 1:**
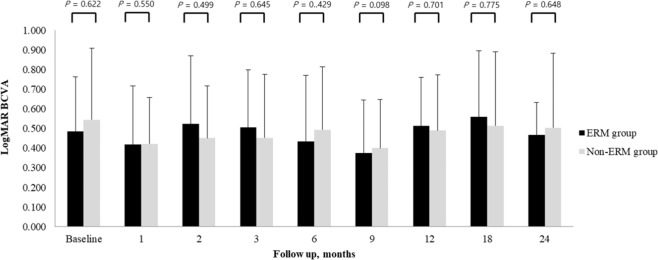
Mean best-corrected visual acuity (BCVA) changes during follow-up of the epiretinal membrane (ERM) and non-ERM groups (Student’ s t-test).

**Figure 2:**
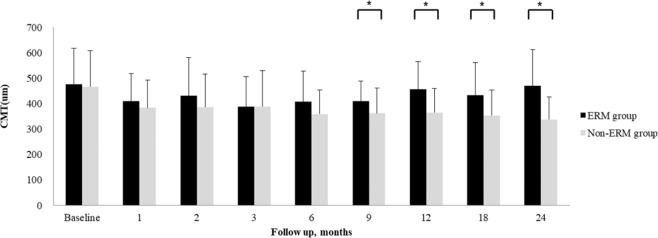
Mean central macular thickness (CMT) during follow-up of the epiretinal membrane (ERM) and non-ERM groups (*P < 0.05, Student’s t-test).

The mean BCVA change was not different between the ERM and non-ERM groups, as shown in Fig. 1. Mean CMT significantly decreased from baseline in all patients at every follow-up visit. However, mean CMT of the ERM group was significantly thicker than that of the non-ERM group after 9 months until 24 months (*P* < 0.05, respectively), as shown in Fig. 2.

In the ERM group, changes of BCVA from pre-injection to 2 weeks post-injection were not different before and after ERM formation, and changes of CMT from pre-injection to 2 weeks post-injection were significantly lower after ERM formation (−62.0 ± 60.9 µm) than before ERM formation (−130.9 ± 93.3 µm) (*P* = 0.005), as shown in Table 3.

**Table 3:** Comparison of mean changes of BCVA and CMT after intravitreal injection before and after epiretinal membrane (ERM) formation in the ERM group.

	Before ERM formation	After ERM formation	*P* value
LogMAR BCVA	0.056 ± 0.116	0.031 ± 0.104	0.400
CMT (μm)	−130.9 ± 93.3	−62.0 ± 60.9	0.005^*^

Values are presented as mean ± standard deviation.

BCVA: best-corrected visual acuity; CMT: central macular thickness.

*P < 0.05, Wilcoxon signed rank test.

### Clinical factors of epiretinal membrane formation

Table 4 shows the results of univariate and multivariate logistic regression analyses of associations between clinical parameters and secondary ERM formation. The number of intravitreal injections (odds ratio [OR] = 1.212, 95% confidence interval [CI] = 1.047 to 1.402, *P* = 0.010) and the use of dexamethasone implant (OR = 1.020, 95% CI = 1.001 to 1.040, *P* = 0.041) were significantly associated with secondary ERM formation following intravitreal injection.

**Table 4:** Univariate and multivariate analysis of risk factors of epiretinal membrane formation.

Parameters	Univariate			Multivariate		
	Odds ratio	CI (95%)	*P*-value	Odds ratio	CI (95%)	*P*-value
Sex, male	0.547	0.174 to 1.719	0.302			
Age at DM diagnosis	1.033	0.984 to 1.084	0.193			
Duration of DM	0.963	0.898 to 1.031	0.280			
Age at DME diagnosis	1.012	0.967 to 1.058	0.608			
DM with CKD	1.673	0.354 to 7.902	0.516			
DM with HTN	3.975	0.855 to 18.480	0.078			
DM with Hyperlipidemia	1.799	0.238 to 13.619	0.570			
Lens status, phakia	1.373	0.402 to 4.693	0.613			
Refraction, S.E (Diopter)	1.043	0.537 to 1.299	0.708			
LogMAR BCVA at initial visit	0.470	0.080 to 2.747	0.402			
IOP at initial visit	0.886	0.751 to 1.044	0.149			
Mean CMT at initial visit	1.001	0.997 to 1.005	0.617			
PRP	0.859	0.282 to 2.611	0.789			
Follow up periods	1.007	0.979 to 1.036	0.619			
Number of intravitreal injections	1.228	1.063 to 1.417	0.005	1.212	1.047 to 1.402	0.010
Agent of intravitreal injection						
Anti-VEGF	0.986	0.970 to 1.002	0.091			
Dexamethasone implant	1.021	1.003 to 1.039	0.019	1.020	1.001 to 1.040	0.041
Triamcinolone	0.996	0.972 to 1.020	0.735			

BCVA: best-corrected visual acuity; CMT: central macular thickness; CKD: chronic kidney disease; DM: diabetes mellitus; DME: diabetic macular edema; IOP: intraocular pressure; PRP: panretinal photocoagulation; S.E: spherical equivalent; VEGF: vascular endothelial growth factor.

## Discussion

It is well known that, among DME patients, there are high incidences of vitreomacular interface abnormality (VMIA), including ERM, vitreomacular attachment (VMA), and vitreomacular traction (VMT) with incomplete posterior vitreous detachment (PVD)^7,11–13^. Although the prevalence of VMIA in DME patients has been shown to vary from 6.5% to 75% according to the diagnostic criteria and methods^7,11,13^, no information has been published about secondary ERM formation after intravitreal injection for DME treatment. Thus, we performed this study specifically investigating secondary ERM among VMIAs based on OCT findings, and we found that 9.5% of eyes developed secondary ERM after intravitreal injection.

The association between intravitreal injection and ERM formation is controversial. Chen *et al*.^9^ reported that platelet-derived growth factor (PDGF) A, VEGF, and their receptors are expressed in ERM and suggested these factors may contribute to ERM progression. They also reported that intravitreal injection of anti-VEGF agent might suppress VMIAs, including ERM formation. On the other hand, there have been reports of secondary ERM formation after intravitreal anti-VEGF injection^14–16^. Anti-VEGF has been reported to cause hypoxia in vascular endothelial cells and increase the expression of connective tissue growth factor (CTGF), which plays an important role in tissue fibrosis and ERM formation^16^. Moreover, Zhang *et al*.^17^ suggested that intravitreal bevacizumab (IVB) injection accelerated fibrosis in PDR patients via the upregulation of fibrosis-related cytokines and reported that IVB exerted pro-fibrotic effects after anti-VEGF therapy *in vitro*. In our study, 6.5% of the eyes that received intravitreal anti-VEGF injection developed secondary ERM, which may have been due to retinal tissue fibrosis after intravitreal anti-VEGF injection.

Currently, intravitreal injection of steroids is used to inhibit the production of inflammatory cytokines and VEGF gene expression in the management of DME^18^. Dexamethasone implant injection, a slow-releasing intravitreal delivery system, has a long-lasting beneficial effect in the treatment of DME. However, the incidence of secondary ERM was significantly higher after intravitreal dexamethasone implant injection than with other agents, and the use of dexamethasone implant is found to be a risk factor for ERM formation in this study. These results are thought to be associated with the drug delivery system. Unlike triamcinolone, dexamethasone implant containing a poly lactic-*co*-glycolic acid (PLGA) matrix was injected into the vitreous cavity using a special mechanical applicator. Meyer *et al*.^19^ reported that dexamethasone implant pellets have a significant muzzle velocity of approximately 0.8 m/s, which decreases exponentially over distance because of the drag force on the vitreous gel. In other words, intravitreal injection of dexamethasone implant may physically induce mechanical stress in the vitreous cavity, including the vitreomacular interface, and lead to VMIA^20^. In addition, it was reported that vitreoproliferative responses, including ERM formation, were observed after intravitreal implantation of a biodegradable PLGA, which comprises the drug vehicle for dexamethasone implant^21^. PLGA matrix that degrades to lactic acid and glycolic acid, and further degrade to carbon dioxide and water induced by hydrolysis^22^. Although, the safety of dexamethasone implant injection has been sufficiently verified, these processes could make alteration of vitreous structures including vitreous liquefaction, posterior vitreous detachment, and lead to vitreoproliferative response. Therefore, dexamethasone implant could be a risk factor of ERM formation after intravitreal injection.

In addition to dexamethasone implant, the analysis of relevant factors found that the incidence of ERM after intravitreal injection also increased as the number of intravitreal injections increased. It is well-known that intravitreal injection itself induces a PVD^23^ and that PVD is closely associated with ERM formation because glial cells and cortical vitreous remnants proliferate in the vitreomacular interface after the occurrence of PVD^24^. Therefore, we thought that frequent intravitreal injections could increase ERM incidence as a result of increased PVD occurrence, making the number of intravitreal injections a risk factor for ERM formation.

We found that BCVA did not increase after ERM formation, and the change of CMT after ERM formation was significantly lower than before ERM formation after intravitreal injection for DME. This means that patients with secondary ERM showed a smaller decrease of CMT after intravitreal injection than before ERM formation, and the effect of intravitreal injection was reduced after ERM formation. This supported the previous report that ERM may act as a physical barrier and decrease drug penetration^10^.

There were several limitations to this study. First of all, we did not evaluate the status of PVD before and after intravitreal injection. However, Ophir *et al*.^25^ previously reported that 52.2% of DME eyes with ERM had incomplete PVD on OCT, suggesting that ERM could appear when incomplete PVD occurred, and ERM could be possible even if complete PVD is not present. Moreover, this study was a retrospective chart review without a control group. This means that it was difficult to evaluate whether the ERM formation occurred as part of the natural course of DME or as a secondary effect after intravitreal injection. We measured BCVA using a Snellen chart and converted to a LogMAR scale instead of using the Early Treatment Diabetic Retinopathy Study (ETDRS) chart as a visual function test. We did not perform more comprehensive visual function tests, such as foveal sensitivity or contrast sensitivity testing. Despite these limitations, it is certain that intravitreal injection itself or dexamethasone implant injection is associated with secondary ERM formation, and the drug effect decreased after secondary ERM formation.

In conclusion, the effect of CMT reduction among DME patient with secondary ERM was significantly less than the effect among patients without ERM following intravitreal injection, and BCVA did not increase. Additionally, we found that the incidence of secondary ERM increased when dexamethasone implant was administered and as the number of intravitreal injections increased. Thus, patients should be informed that intravitreal injection for the treatment of DME may cause secondary ERM formation and that the effects of intravitreal injections may decline after ERM formation.

## Methods

Medical records were retrospectively reviewed after approval of the Institutional Review Board of Kyungpook National University Hospital (IRB No. 2018-11-010). The review was conducted in accordance with the tenets of the Declaration of Helsinki. Written informed consent was obtained from all participants before study enrollment.

We included patients who had DR with DME aged over 18 years and who received at least 1 intravitreal injection of anti-VEGF, triamcinolone acetonide, or dexamethasone implant and were followed up for more than 24 months from January 2012 to December 2016. Patients received intravitreal injection of anti-VEGF or steroid followed a pro re nata (PRN) regimen, but ranibizumab, aflibercept and dexamethasone implant were injected according to health care insurance^26–29^. Patients were excluded if they met any of the following criteria: (1) the presence of uveitis or retinal vein occlusion that may cause macular edema; (2) history of procedures, such as neodymium:yttrium–aluminum–garnet (Nd:YAG) posterior capsulotomy or phacoemulsification that can cause macular edema within 1 month before intravitreal injection; (3) complications of DR other than DME, such as vitreous hemorrhage or tractional retinal detachment; (4) previous vitreoretinal surgery or glaucoma surgery; (5) the presence of media haze that may affect fundus examination and OCT examination; and (6) uncontrolled diabetes mellitus (serum HbA1c > 10%).

DME was documented by fundus examination and spectral-domain OCT (SD-OCT, Spectralis®, Heidelberg Engineering, Heidelberg, Germany). DME was defined as retinal thickening of more than 1 disc diameter, diffuse or cystoid changes within half of a disc diameter of the fovea and CMT > 300 μm on OCT. The type of DME was classified as diffuse macular edema, cystoids macular edema and serous retinal detachment according to Otani *et al*.^30^. ERM was defined as a highly reflective membranous structure at the vitreomacular interface on OCT^24^. OCT image interpretation including detection of ERM was performed by one physician (YKK).

All intravitreal injections were performed under sterile conditions in an operating room. Eyes were anesthetized with topical 0.5% proparacaine hydrochloride (Paracaine®, Hanmi, Seoul, Korea), and 5% povidone-iodine was used for prophylaxis of endophthalmitis. Subsequently, intravitreal injection of an anti-VEGF agent, for example bevacizumab (Avastin®, Genentech, South San Francisco, California, USA) 1.25 mg/0.05 mL, ranibizumab (Lucentis®, Genentech, South San Francisco, California, USA) 0.5 mg/0.05 mL, or aflibercept (Eylea®, Regeneron, Tarrytown, NY, USA) 2.0 mg/0.05 mL was administrated at 3.5 to 4.0 mm posterior to the limbus using a 30-gauge needle. Triamcinolone (Triam®, Shinpoong, Seoul, Korea) 4.0 mg/0.1 mL or dexamethasone implant (Ozurdex®, Allergan, Irvine, CA, USA) were also administrated at the par plana. Topical 0.5% levofloxacin (Cravit®, Santen, Osaka, Japan) eye drops were prescribed 4 times daily for 1 week after intravitreal injection.

Ophthalmic examinations were performed before and 2 weeks after every intravitreal injection, including BCVA using a Snellen chart, intraocular pressure (IOP), slit lamp examination, fundus examination, and SD-OCT examination. BCVAs were converted to the LogMAR for statistical analysis. All examinations were repeated after 1, 2, 3, 6, 9, 12, 18, and 24 months after the first intravitreal injection. The number and type of intravitreal injection were obtained from operation records.

Statistical analyses were performed using SPSS Statistics version 20 (IBM Corp., Armonk, NY, USA). Repeated measures analysis of variance (ANOVA) corrected by the Bonferroni method was used to compare the mean BCVA and CMT according to the follow-up periods. Student’s t-test was performed to compare the mean BCVA and CMT in both groups. In patients with secondary ERM, Student’s t-test and one-way ANOVA were used to compare the incidence of ERM formation according to clinical factors. The Wilcoxon signed-rank test was used to compare BCVA and CMT changes between pre-injection and 2 weeks post-injection before and after ERM formation. Univariate logistic regression analysis was performed to calculate risk factors of secondary ERM. Variables with a *P* value < 0.05 in the univariate analysis were included in subsequent multivariate analysis. For all statistical tests, a *P* value < 0.05 was considered significant.
